# The identity of the tropical African *Polichne mukonja* Griffini, 1908 (Orthoptera, Tettigoniidae, Phaneropterinae)

**DOI:** 10.3897/zookeys.621.9725

**Published:** 2016-10-03

**Authors:** Bruno Massa

**Affiliations:** 1 Department of Agriculture and Forest Sciences, University of Palermo, Viale Scienze 13, 90128 Palermo, Italy

**Keywords:** Catoptropterigini trib. n., distribution, Griffinipteryx gen. n., taxonomy, tropical Africa

## Abstract

*Polichne mukonja* Griffini, 1908 from Cameroon was hitherto known only from the holotype preserved at the Royal Belgian Institute of Natural Sciences, Brussels. This was probably due to the fact that the genus *Polichne* Stål, 1874 distributed only in Australia and Papua New Guinea. In view of this distribution, the tropical African species was therefore overlooked in the African literature. The recent discovery of two specimens at the Naturhistorisches Museum, Vienna, now provides us with a better understanding of the identity of this taxon, which is related to the African genus *Catoptropteryx* Karsch, 1890. *Polichne mukonja* is here transferred to a new genus *Griffinipteryx* and both taxa are proposed to be included in the new tribe Catoptropterigini.

## Introduction

Griffini (1908), when describing *Polichne mukonja* from Cameroon (Mukonje Farm), highlighted that it was the first African species from this genus. The genus *Polichne* Stål, 1874 was indeed previously known only from Australia and Papua New Guinea. However, it seemed very probable that the species described by Griffini belonged to a different genus from *Polichne*, but the detailed description by Griffini (1908), has been forgotten for more than a century. Curiously, Ragge (1968, 1980) also overlooked it, first when he listed all the African Phaneropterinae (1968) and then when revising the African Phaneropterinae with open tympana (1980).

A recent study of the material preserved at the Naturhistorisches Museum of Vienna revealed two specimens, both females (like the holotype). These were probably collected towards the end of 1800s, prior to the description of the species by Griffini (1908). These specimens possess the following characters: slender body, fore coxae armed, open tympana on both sides of fore tibiae, black stripe along the body, and very short ovipositor, similar to that of *Catoptropteryx* Karsch, 1890. They fit very well with the description by Griffini (1908) of *Polichne mukonja*. Jerome Constant (RBINS, Royal Belgian Institute of Natural Sciences, Brussels) kindly provided a photograph of the type specimen; later this was compared with the Vienna specimens and it was possible to establish that they both belonged to the species described by Griffini (1908). However, on the base of its characters, it is now possible to ascertain that the African species *Polichne mukonja* belongs to an entirely new genus to which its description is being presented in this work.

## Material and methods

The material examined (see below) is preserved at the Naturhistorisches Museum of Vienna (NMW). This study of *Polichne mukonja* has been carried out on specimens belonging to genera considered within the Ephippithytae group, particularly in the shape of the pronotum, the face and ovipositor and the presence of spines on the femora. Measurements were taken using a caliper Digimax measy 2000 (precision: 0.01 mm). The following measurements were taken (in mm): Body length: dorsal length from the head to the apex of the abdomen, ovipositor excluded; Pronotum length: length of the pronotum along dorsal median line; Pronotum height: maximum height of the pronotum; Hind femur: length of hind femur; Tegmina: length of tegmina; Ovipositor: maximum length from the subgenital plate to the tip of the ovipositor. Photographs were taken with a Nikon Coolpix 4500 digital camera, mounted on a Wild M5 Stereomicroscope, and photos were integrated using the freeware CombineZP (Hadley 2008).

## Results and discussion

### Griffinipteryx gen. n.

Taxon classificationAnimaliaOrthopteraTettigoniidae

http://zoobank.org/BA4B0B72-6445-4E17-9D2C-841C5EA3348B

Figs 1–5 , 6

#### Type species.

*Griffinipteryx mukonja* (= *Polichne mukonja* Griffini, 1908).

#### Diagnosis.

**Figures 1–5.**
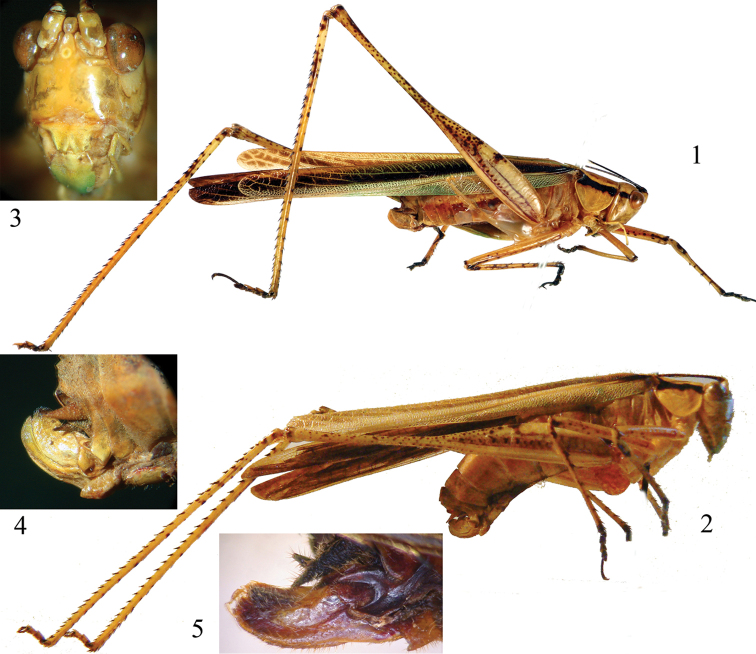
**1** The Holotype of *Polichne mukonja* Griffini, 1908, now included in the new genus *Griffinipteryx* (photo by Jerome Constant, Royal Belgian Institute of Natural Sciences, Brussels) **2** *Griffinipteryx mukonja*, female from Cameroon (Naturhistorisches Museum, Vienna) **3** Face in frontal view of the same **4** Lateral view of the ovipositor of *Griffinipteryx mukonja* **5** Lateral view of the ovipositor of *Catoptropteryx extensipes* Karsch, 1896.

**Figures 6–8.**
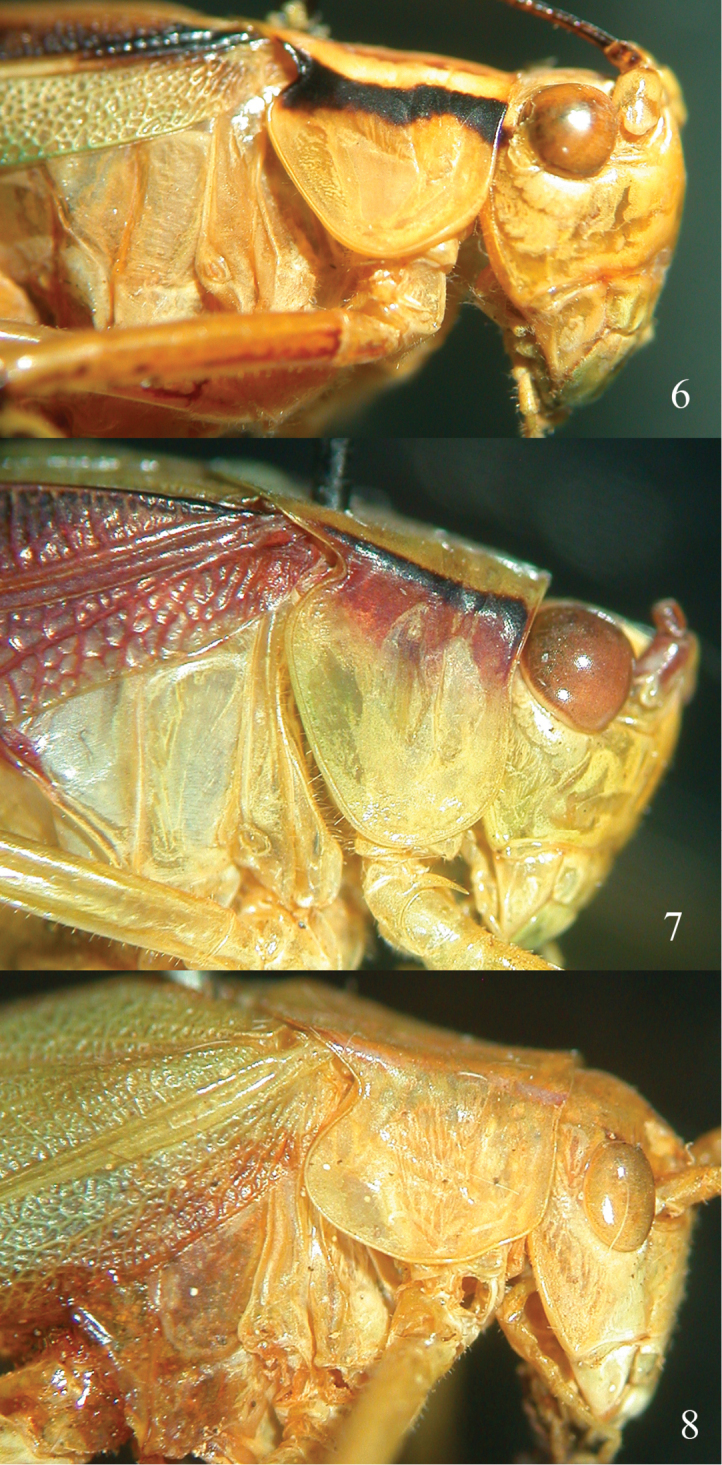
**6** Lateral view of head and pronotum of *Griffinipteryx mukonja* from Cameroon **7** Lateral view of head and pronotum of *Catoptropteryx punctulata* Karsch, 1890 **8** Lateral view of head and pronotum of *Polichne parvicauda* (Stål, 1861), type species of the genus *Polichne*.

*Griffinipteryx* is characterized by slender body, lateral lobes of pronotum as deep as wide, ovipositor much reduced (Figs 1–4, 6).

#### Description.

Head and antennae: fastigium of vertex narrow and pointed, much narrower than the first antennal segment. Eyes round, prominent, face smooth without fronto-genal carinae, higher than wide.

*Thorax*: pronotum just longer than high, with a well-developed humeral excision, lateral lobes are as deep as wide.

*Legs*: long (ratio body length/length hind femur: 0.9), fore coxae armed with a fine spine, fore, mid and hind femora unarmed, fore, mid and hind tibiae with ventral and dorsal spines. Fore tibiae with anterior and posterior open tympana.

*Tegmina* elongated, well developed, shorter than hind wings.

Ovipositor very reduced, crenulated on upper apex.

#### Etymology.

*Griffinipteryx* (Griffini + pteryx) is dedicated to the late Achille Griffini (1870–1932), distinguished Italian entomologist, who studied many African collections of Orthoptera and described *Polichne mukonja*; the Greek suffix *pteryx* (wing) is a reminder of the genus *Catoptropteryx*.

#### Affinities.

*Catoptropteryx* is certainly the only other African genus related to *Griffinipteryx*. In particular, some *Catoptropteryx punctulata* Karsch, 1890 specimens have a similar colour pattern in the pronotum (Fig. 7), even if the black stripe is absent on the lateral lobes. *Catoptropteryx* may have small spines at the ventral inner margin of fore, mid and hind femora, while *Griffinipteryx* gen. n. has unarmed fore and mid femora (in actual fact a very small spine is present at the base of the ventral inner margin of the fore and on the ventral outer margin of the mid femora). Other differences are also detectable in the shape of the lateral lobes of the pronotum. In *Catoptropteryx* the pronotal lobes are deeper than wide, while in *Griffinipteryx* gen. n. they are as deep as wide (Figs 6–7). Also the length of the hind femora is proportionally longer in *Griffinipteryx* gen. n. than in *Catoptropteryx*. However, the most important character that separates the two genera is the shape of the ovipositor. While in *Catoptropteryx* the ovipositor is very reduced and simplified (cf. Fig. 2 of Huxley 1970), in *Griffinipteryx* gen. n. it is more apically chitinous, mainly in the apical dorsal margin, where it is a somewhat crenulated. In some species of *Catoptropteryx* the dorsal valve may also be crenulated (Huxley 1970), but the ovipositor appears longer with slender infra- and supra-gonangulum, and less chitinous. In *Griffinipteryx* the ovipositor and the subgenital plate are more chitinous and infra- and supra-gonangulum are stout (compare Figs 4 and 5).

### Griffinipteryx mukonja

Taxon classificationAnimaliaOrthopteraTettigoniidae

(Griffini, 1908)

#### Depository of the type.

Cameroon, Mukonje Farm (♀ holotype) (RBINS, Brussels) (photograph examined by courtesy of Jerome Constant).

#### Material examined.

Cameroon, Johann-Albrechtshöhe [near Lake Barombi, ca. 80 km from Mukonje], Rhode (2♀) (coll. Brunner von Wattenwyl, NMW, Vienna).

#### General habitus and colour.

Yellow-brownish, a black stripe from behind the eye on the head through the lateral lobes of the pronotum and gradually broadening along the tegmina. Abdomen yellow with some brown spots; legs yellow with many black dots; tympanum with a black anterior margin; fore and mid tarsi black, hind tarsi yellow with a black base.

#### Description.

Female. Fastigium of vertex sulcate above; face smooth without fronto-genal carinae, higher than wide. Pronotum without lateral carinae, surface shiny, with a well-developed humeral excision on the lateral lobes. Anterior margin of pronotum straight, posterior margin gently rounded, pronotum lobes rounded on posterior margins. Fore coxae armed with a fine spine; a very small spine is present at the base of the ventral inner margin of fore and of ventral outer margin of the mid femora, hind femora unarmed; fore tibiae with 3 black spines on ventral inner margin, 3 spines + 1 apical spur on ventral outer margin, 1 spine dorsal on fore tibia above tympanum + 1 apical spur; mid tibiae with 4-5 black spines on ventral inner margin + 1 apical spur, 6 black spines ventral on outer margin + 1 apical spur, 1 dorsal inner black spine + 1 apical spur; hind tibiae with 9-10 black spines ventral on both margins + 2 apical spurs on each side; dorsal margins of hind tibiae with many black and yellow black tipped spines + 1 apical spur on each side. Abdomen: styli pointed, ovipositor very short with crenulated dorsal apex. Subgenital plate triangular and short, as long as wide, apically rounded.

Male: unknown.

#### Measurements.

See Table 1.

**Table 1.** Measurements of two females of *Griffinipteryx mukonja* (Griffini, 1908); measurements in brackets recorded by Griffini (1908).

Body length	22.7–24.5 (20)
Pronotum length	4.8–4.9 (4.7)
Pronotum height	4.3–4.4
Length of hind femur	25.8–26.0 (26.5)
Tegmina length	27.5–29.2 (27)
Tegmina width	3.4 (3.7)
Length of hind wing	31.8–33.5 (31.3)
Ovipositor	3.2–3.3 (2.4)

### Catoptropterigini trib. n.

Taxon classificationAnimaliaOrthopteraTettigoniidae

http://zoobank.org/7A9E56A9-2D0C-41C7-88F1-A6E836D84276

#### Type genus.

*Catoptropteryx* Karsch, 1890.

Currently the genus *Catoptropteryx* Karsch, 1890 belongs to the species group Ephippithytae Brunner von Wattenwyl, 1878, together with another eleven genera found in Australia and Papua New Guinea. This group of species is very heterogeneous and probably the sole character that brings them together is the reduction of the female ovipositor, even if its structure is much different in some of them. To include the African genus *Catoptropteryx* in this heterogeneous group of Australasian genera seems like a biogeographical nonsense. As a first step, the comparison of the African species *Polichne mukonja* described by Griffini (1908) was carried out on the type species *Polichne parvicauda* (Stål, 1861) (♀ from Australia, NMW), originally described as *Phaneroptera*, and the following differences were noticed (Fig. 8): presence of fronto-genal carinae, eyes oval, fastigium sulcate, short ovipositor, but not as reduced as in *mukonja*, pronotum with a deep humeral excision, just longer than high, of different shape compared to *mukonja*. Then, the type species of the genus *Ephippitytha*^1^ Serville, 1838 [*Ephippithyta trigintiduoguttata* (Serville, 1838)] was examined (6 specimens of both sexes from Australia, NMW). It has clear fronto-genal carinae, large spines on both margins of the hind femora, one spine on both apices of fore, mid and hind femur knees, pronotum centrally narrowed, similar to a saddle, ovipositor much reduced, but slender and pointed. In addition, the ovipositor of the other nine genera in the Ephippithytae group is heterogeneous even if reduced, eyes may be round or elongate and fronto-genal carinae may be both present or absent.

When the first species of *Catoptropteryx* were discovered, they were described within the Australian genus *Caedicia* Stål, 1874 (e.g.: *afra*: Karsch 1888; *apicalis*: Bolívar 1893), also included in the Ephippithytae group. Thus, when Karsch (1890) erected for them the genus *Catoptropteryx* it was considered logical at the time to include this genus in the Ephippithytae group, erected by Brunner von Wattenwyl in 1878. However, also *Caedicia* is evidently different from *Catoptropteryx* (examined 6♂ of the type species of the genus, *Caedicia pictipes* Stål, 1874, and a few specimens of the other six species, *Caedicia marginata* Brunner von Wattenwyl, 1878, *Caedicia concisa* Brunner von Wattenwyl, 1878, *Caedicia septentrionalis* Brunner von Wattenwyl, 1878, *Caedicia simplex* (Walker, 1869), *Caedicia inermis* Brunner von Wattenwyl, 1878, and *Caedicia scalaris* Brunner von Wattenwyl, 1878, all from Australia, NMW, coll. Brunner von Wattenwyl). The characteristics of this genus are: eyes round, small spines on femur knees, presence of spines on lower margins of femora, fronto-genal carinae. While these characters are evident in the type species, they are not always present in other species (e.g.: eyes may be oval, spines in femur knees may be absent). Overall, it may be confirmed that the Ephippithytae group is very heterogeneous and therefore it cannot be considered as a tribe.

Presently, in the light of the revision of the genus *Catoptropteryx* by Huxley (1970) and the discovery of the identity of the African *Polichne mukonja* Griffini, 1908 (now *Griffinipteryx mukonja*), the new tribe Catoptropterigini seems a logical taxonomical consequence.

Characters of the tribe are the following. Fastigium narrower than first antennal segment, furrowed, face smooth without fronto-genal carinae, eyes round, very small spines or unarmed lower margins of fore and mid femora, hind femora with few small spines or unarmed, tegmina longer than wings, ovipositor very reduced.

### Concluding remarks

In 1908 the Italian entomologist Achille Griffini described the katydid *Polichne mukonja* from Cameroon. Incomprehensibly he described this taxon within the Australian genus *Polichne*, which does not show any morphological affinities with it. Only the holotype was known (specimen preserved at the Royal Belgian Institute of Natural Sciences, Brussels), and later on this taxon was no longer cited. When an opportunity arose to study the African material preserved in the Naturhistorisches Museum of Vienna, during a Synthesys project, two other specimens of this taxon were discovered and it was possible to understand its identity. *Polichne mukonja* resulted as belonging to a newly established genus (*Griffinipteryx*) and is rather related to the tropical African genus *Catoptropteryx*, for which the new tribe Catoptropterigini is here proposed. This present work continues to demonstrate that Natural History museums preserve interesting treasures that are still waiting to be discovered.

## Supplementary Material

XML Treatment for Griffinipteryx

XML Treatment for Griffinipteryx mukonja

XML Treatment for Catoptropterigini

## Footnotes

1
*Ephippithyta* Brunner von Wattenwyl, 1878 is a synonym.
